# Reclassification of *BRCA2* Variants of Uncertain Significance Using Saturation Genome Editing Combined with Clinical Phenotypes in Breast Cancer

**DOI:** 10.3390/curroncol33090521

**Published:** 2026-08-31

**Authors:** Yueran Shen, Jiuan Chen, Li Hu, Jie Sun, Juan Zhang, Lu Yao, Ye Xu, Yuntao Xie

**Affiliations:** Familial & Hereditary Cancer Center, Key Laboratory of Carcinogenesis and Translational Research (Ministry of Education/Beijing), Peking University Cancer Hospital & Institute, Beijing 100142, China; shenyueran001@126.com (Y.S.); cja_3303@163.com (J.C.); whdxhl@163.com (L.H.); sunjieelro@bjmu.edu.cn (J.S.); zhangjuan100@126.com (J.Z.); yaolu@bjmu.edu.cn (L.Y.)

**Keywords:** breast cancer, *BRCA2*, variants of uncertain significance, saturation genome editing, clinical phenotype

## Abstract

Purpose: To evaluate the pathogenicity of *BRCA2* variants of uncertain significance (VUS) located within the functionally critical exons 15–26 using published SGE data, and to reclassify these VUS by integrating clinical phenotypes. Methods: A total of 15,092 breast cancer patients were enrolled in this study, among which 457 distinct *BRCA2* VUS were identified in 1051 carriers. Based on SGE scores, 88 *BRCA2* VUSs within exons 15–26 were functionally assessed and carriers reclassified as functionally pathogenic, functionally benign, or remaining VUS. Clinicopathological characteristics were subsequently compared across variant groups. Results: Of these 88 evaluated *BRCA2* VUSs (187 carriers), 15 were reclassified as functionally pathogenic (20 carriers), 66 as functionally benign (154 carriers), and 7 remained VUS (13 carriers). Compared with non-carriers, carriers with functionally pathogenic variants exhibited a significantly higher prevalence of a family history of any cancer (65.0% vs. 31.1%, *p* = 0.002), particularly breast and/or ovarian cancer (35.0% vs. 10.0%, *p* = 0.002), as well as a trend toward a higher incidence of bilateral breast cancer (10.0% vs. 2.4%, *p* = 0.085). In contrast, individuals harboring functionally benign variants demonstrated clinicopathological characteristics similar to non-carriers. Conclusion: SGE-based functional scoring system provides a reliable approach for reclassifying *BRCA2* VUS. When integrated with clinical phenotypes, it enhanced the accuracy of pathogenicity assessment.

## Simple Summary

Genetic testing for breast cancer often finds changes in the *BRCA2* gene that have unknown effects, making it difficult to guide patient management. In this study of 15,092 breast cancer patients, we used published saturation genome editing (SGE) results, combined with clinical and family history data, to assess 88 uncertain variants in exons 15–26 of *BRCA2*. We found that 15 variants were functionally pathogenic and associated with stronger family histories of cancer, particularly breast and ovarian cancers, whereas 66 variants were functionally benign. These findings suggest that combining SGE with patient characteristics can improve *BRCA2* variant interpretation. This approach may support future genetic counseling, cancer risk assessment, and variant classification guidelines, although further validation in larger and more diverse populations is needed.

## 1. Introduction

*BRCA2* is a well-established breast cancer (BC) susceptibility gene that plays a critical role in DNA damage repair [1,2,3]. It encodes an essential component of the homologous recombination pathway, which mediates the repair of DNA double-strand breaks and maintains genomic stability [4,5]. Pathogenic germline variants in *BRCA2* significantly increase the risk of breast cancer and represent a major genetic cause of hereditary breast cancer [6,7,8,9,10]. Notably, patients with *BRCA*-deficient tumors often benefit from poly (ADP-ribose) polymerase (PARP) inhibitors [11,12,13]. Therefore, accurate classification of *BRCA2* variants is essential for risk assessment, individualized treatment selection, and clinical management, including genetic counseling [14,15,16].

As genetic testing becomes widely applied, an increasing number of *BRCA2* variants of uncertain significance (VUS) are being identified [17,18]. This uncertainty greatly limits their clinical utility and complicates evidence-based decision-making [19]. According to the ClinVar database, 13,201 single-nucleotide variants (SNVs) have been reported in *BRCA2*, of which approximately 59% are categorized as variants of uncertain significance [20]. Recently, saturation genome editing (SGE) has been applied to systematically evaluate more than 6000 variants within the functionally critical regions encoded by using SGE functional scores from those studies [20,21]. This high-throughput functional approach provides an objective framework for resolving the clinical ambiguity of *BRCA2* VUS and offering a promising strategy [21].

In the present study, we reclassified *BRCA2* VUS identified in a large hospital-based breast cancer cohort using SGE functional scores. By integrating comprehensive clinicopathological data, we further characterized the clinical features of patients harboring reclassified functionally pathogenic variants, in comparison with non-carriers and carriers of known pathogenic *BRCA2* variants.

## 2. Materials and Methods

### 2.1. Patients and Sequencing Data

Data from a total of 15,092 patients treated at Peking University Cancer Hospital were included in this study, consisting of 13,781 consecutive breast cancer patients from October 2003 to November 2018, and 1311 breast cancer patients from December 2018 to August 2021. All patients underwent *BRCA2* variant screening using Sanger sequencing and/or multigene panel sequencing [22]. Products were sequenced on a HiSeq 2500 (Illumina, San Diego, CA, USA) to at least an average depth of 200-fold coverage (BGI Genomics Co., Ltd., Beijing, China or Novogene Co., Ltd., Beijing, China). Variants were annotated in accordance with the Human Genome Variation Society (HGVS) nomenclature guidelines, based on the National Center for Biotechnology Information (NCBI) reference sequence NM_000059.4 [23,24]. Family history and clinicopathological data were obtained through telephone interviews and/or extracted from medical records. This study was approved by the Research and Ethical Committee of Peking University Cancer Hospital, and written informed consent was obtained from all participants.

### 2.2. Variant Classification

Following the guidelines of the American College of Medical Genetics and Genomics and the Association for Molecular Pathology (ACMG/AMP) [25], we systematically classified *BRCA2* variants from 15,092 patients. Truncating variants (nonsense and frameshift variants) were classified as pathogenic only if they were predicted to undergo nonsense-mediated mRNA decay (NMD), consistent with ACMG/AMP PVS1 criteria. All other variants were considered pathogenic or likely pathogenic based on ClinVar database (http://www.ncbi.nlm.nih.gov/clinvar/, accessed on 30 May 2025) and/or reports the literature. Variants categorized as benign or likely benign in ClinVar were classified as benign. All remaining variants were considered variants of uncertain significance (VUSs). Patients carrying more than one variant were assigned to the highest-risk category according to the most clinically consequential alteration. For statistical analysis, non-carriers were defined as individuals with benign/likely benign variants or no identified variants whatsoever.

### 2.3. Variants Reclassification by Saturation Genome Editing

The pathogenicity of *BRCA2* VUSs identified in this cohort of breast cancer patients was evaluated using functional scores derived from saturation genome editing (SGE), as previously reported [20,21]. To ensure analytical rigor, a variant was classified as functionally pathogenic only if concordant pathogenic classifications were obtained from two independent SGE studies of *BRCA2*. Similarly, a variant was classified as functionally benign only if both studies consistently indicated a benign functional impact. Variants with discordant classifications between the two studies were retained as variants of uncertain significance (VUS). For variants reclassified in only one study and not evaluated in the other, the available classification result was used.

### 2.4. Definition of Variable

Definitions of study variables, including family history of malignant tumor, family history of breast cancer, tumor size, estrogen receptor (ER)/progesterone receptor (PR)/human epidermal growth factor receptor 2 (HER2) status, tumor grade, and lymph node status have been described in our previous studies [26]. Age at breast cancer diagnosis was defined as the patient’s age at initial diagnosis. Bilateral BC was defined as the occurrence of contralateral primary BC, either synchronously or metachronously.

### 2.5. Statistical Analysis

Categorical variables were compared using the χ^2^ test or Fisher’s exact test, as appropriate. Continuous variables were analyzed using independent t-tests. A *p*-value < 0.05 was considered statistically significant. The Bonferroni correction was applied to account for multiple comparisons, with the corrected significance threshold noted in the table legend for reference. All statistical analyses were performed using R 4.3.2.

## 3. Results

### 3.1. BRCA2 Variants of Uncertain Significance in Breast Cancer Patients

**Figure 1:**
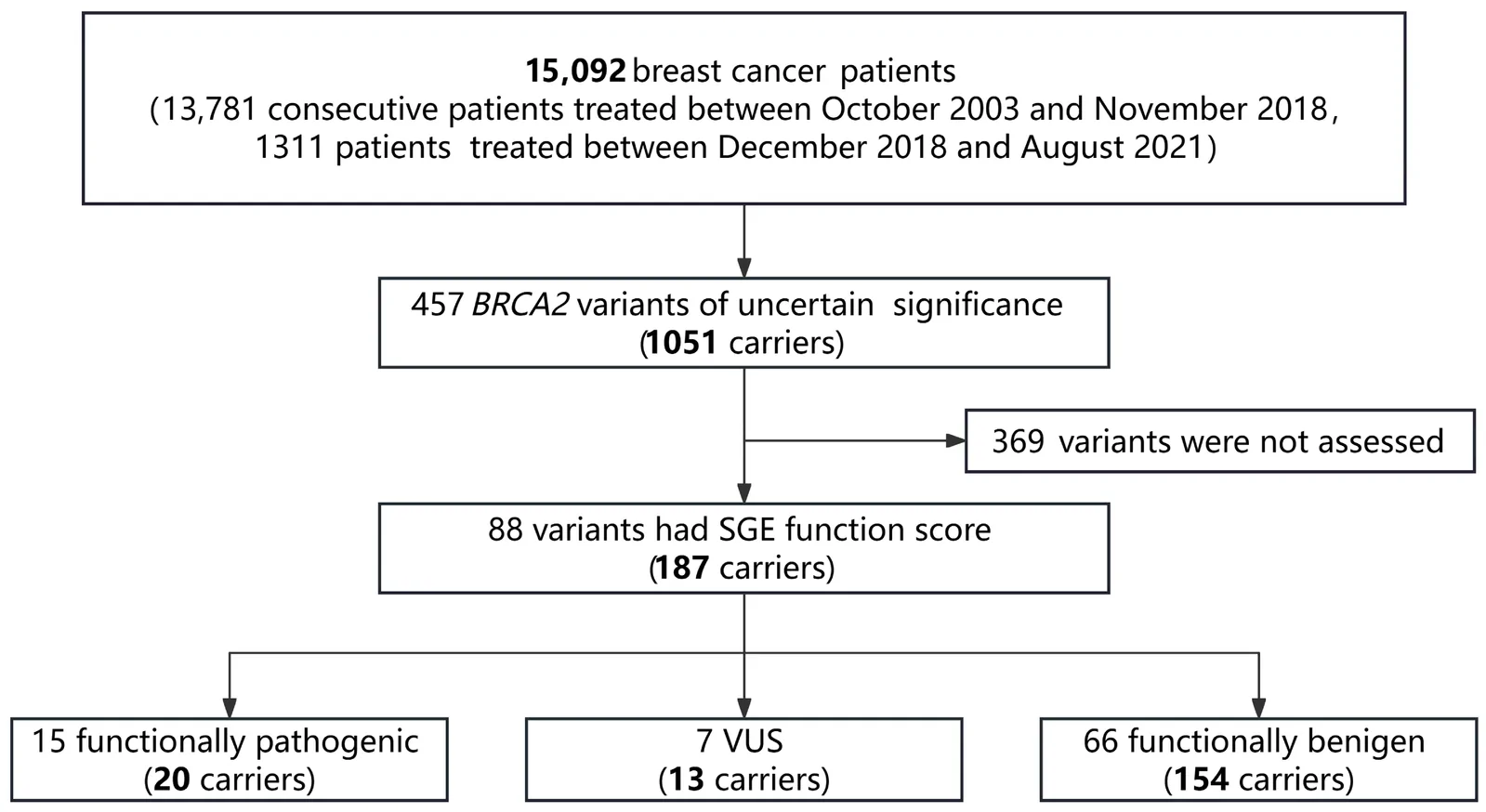
Classification of *BRCA2* variants of uncertain significance found in 15,092 breast cancer patients according to saturation genome editing function score. VUS, variant of uncertain significance.

**Figure 2:**
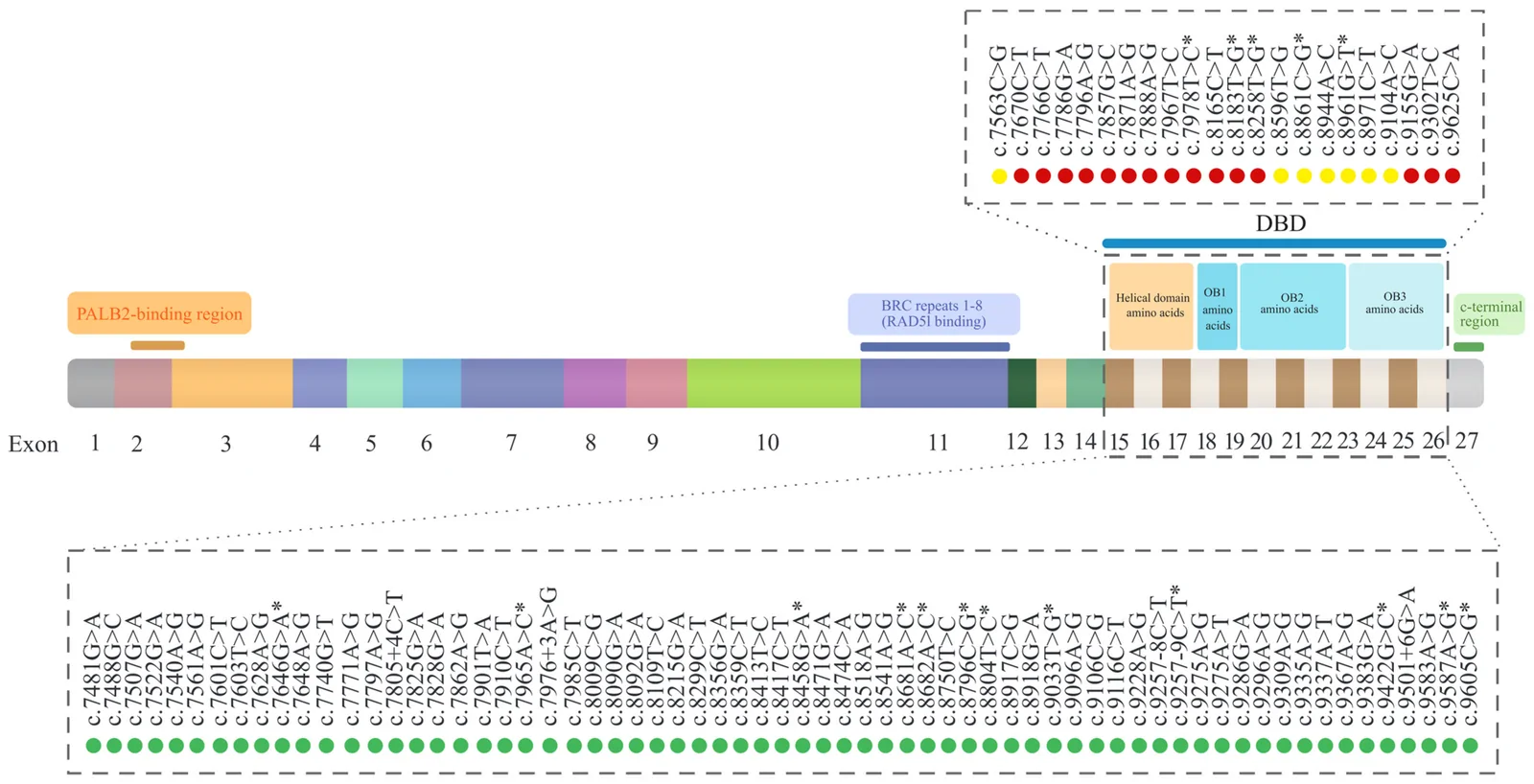
Schematic of *BRCA2* protein domains and distribution of variants classified by saturation genome editing (SGE). Red dots represent variants reclassified as functionally pathogenic, yellow dots represent variants that remained variants of uncertain significance, and green dots represent variants reclassified as functionally benign. * Indicates novel variants that had not been reported in disease databases (the deadline for ClinVar is 30 May 2025). DBD, DNA-binding domain; OB, Oligonucleotide/oligosaccharide-binding fold.

Among the 15,092 patients, 316 pathogenic or likely pathogenic variants were identified in 506 carriers, 162 benign or likely benign variants in 13,395 carriers (per ClinVar annotations), and 140 patients had no identifiable *BRCA2* mutations. In addition, 457 *BRCA2* variants of uncertain significance (VUS) were identified in 1051 patients. Of these VUSs, 88 variants located within exons 15–26 (identified in 187 carriers) were classifiable based on SGE functional scoring criteria, as they were the only VUSs from our cohort that were also evaluated in the two published SGE studies (Table 1) [20,21]. These 88 VUSs were further categorized into three categories: functionally pathogenic (*n* = 15; 20 carriers), VUS (*n* = 7; 13 carriers), and functionally benign (*n* = 66; 154 carriers) (Figure 1 and Figure 2). Data for each individual variant, including population frequency and computational functional predictions, are summarized in Supplementary Table S1.

### 3.2. Clinical Phenotypes of BRCA2 VUS Carriers Stratified by SGE Results

Compared with non-carriers, patients harboring functionally pathogenic variants showed a significantly higher prevalence of a family history of malignant tumor (65.0% versus 31.1%, *p* = 0.002), particularly breast cancer and/or ovarian cancer (35.0% vs. 10.0%, *p* = 0.002). In addition, carriers with functionally pathogenic variants had a higher incidence of contralateral breast cancer than non-carriers (10.0% versus 2.4%, *p* = 0.085) (Table 2). In contrast, patients with functionally benign variants exhibited no significant differences in clinicopathological characteristics compared with non-carriers (Table 2).

### 3.3. Reclassification of BRCA2 VUSs Based on SGE and ACMG

We further integrated SGE functional scores with ACMG criteria to reclassify these 88 VUSs into five categories: pathogenic, likely pathogenic, VUS, likely benign, and benign. Of these 88 reclassified variants, 6 were reclassified as pathogenic (9 carriers), 18 as VUS (27 carriers), and 64 as benign (151 carriers). A total of 11 variants showed discrepant classifications between SGE alone and the combined SGE-ACMG framework (Supplementary Tables S2 and S3).

### 3.4. Pedigrees of Functionally Pathogenic Variants

Among the 20 carriers of functionally pathogenic *BRCA2* variants, 13 patients had a family history of malignant tumors, including 7 with a family history of breast cancer or ovarian cancer. Detailed telephone follow-up was conducted for 9 families to construct comprehensive pedigrees.

**Figure 3:**
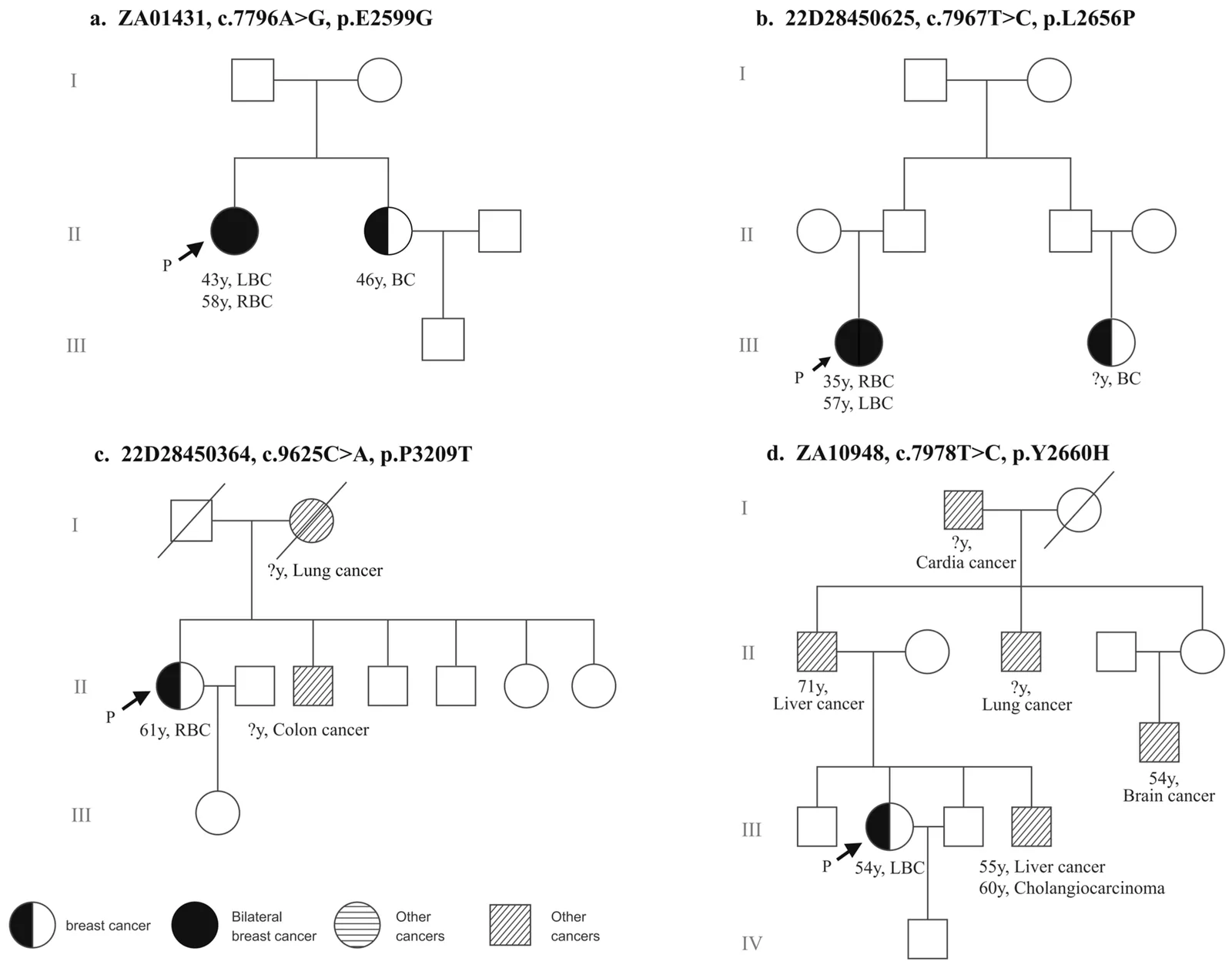
Pedigrees of probands carrying functionally pathogenic variants. Pedigrees were collected for four probands ((**a**), ZA01431, (**b**), 22D28450625, (**c**), 22D28450364 and (**d**), ZA10948). In each pedigree, the degree of relationship is indicated on the left, and the age at cancer diagnosis and current age, where applicable, are shown below each symbol. Circles and squares represent females and males, respectively. A diagonal line through a symbol indicates that the individual is deceased. The letter “P” and an arrow identify the proband in each family. Roman numerals indicate the generations in each pedigree. BC, breast cancer; LBC, left breast cancer; RBC, right breast cancer.

Patient ZA01431 (*c.7796A>G*) was diagnosed with left-sided BC at age 43 and contralateral BC at 58; her sister was also diagnosed with BC at age 46. Patient 22D28450625 (*c.7967T>C)* developed right-sided BC at age 35 and contralateral BC at 57, with a first-cousin diagnosed with BC (age at onset unknown). Additionally, patients 22D28450364 (*c.9625C>A*) and ZA10948 (*c.7978T>C*) had had family histories of multiple other cancer types. The complete pedigrees of these well-characterized families are shown in Figure 3. Five additional carriers—23D04591198 (*c.9302T>C*), M32–17210 (*c.7857G>C*), 24D04024913 (*c.9625C>A*), ZA02478 (*c.7888A>G*), and 22D28449761 (*c.8165C>T*)—had BC family history involving first-degree relatives (mothers or sisters). Collectively, these familial clustering events provide supporting evidence for the pathogenicity of these eight functionally pathogenic variants.

## 4. Discussion

In this study, we applied saturation genome editing (SGE) [20,21] functional scores to reclassify 88 *BRCA2* variants of uncertain significance identified in a large real-world cohort of 15,092 patients with breast cancer. Functional assessment classified 15 variants as functionally pathogenic and 66 as functionally benign, while 7 remained unresolved. Notably, patients with functionally pathogenic *BRCA2* variants exhibited significantly higher prevalence of a family history of cancer, particularly breast cancer and ovarian cancer, as well as an increased incidence of contralateral breast cancer. These findings align with the established phenotypic spectrum of hereditary BC and support the clinical validity of SGE-based functional classification in *BRCA2* [27,28].

A major challenge in clinical genetic testing is the high proportion of VUS in *BRCA*, the majority of which are missense variants [14,29]. Conventional interpretation strategies, which depend on in silico prediction, population frequency, and evolutionary conservation, often provide insufficient or conflicting evidence, thereby limiting their clinical utility [30]. SGE addresses these limitations by introducing variants into their endogenous genomic context and quantitatively measuring their functional impact at single-nucleotide resolution [31,32]. This high-throughput approach enables systematic and reproducible functional annotation across large genomic regions [32], providing quantitative evidence that can directly inform variant classification within established frameworks such as ACMG/AMP.

Consistent with prior studies in *BRCA1* and other homologous recombination genes [22,31,33], our findings demonstrate that SGE-based functional classification reliably distinguishes pathogenic from benign variants and correlates with clinically relevant phenotypes. Our previous work in *BRCA1* showed that loss-of-function variants were associated with more aggressive clinicopathological features [22]. Here, we extend these observations to *BRCA2* and demonstrate a similar concordance between functional classification and clinical presentation. Together, these cross-gene data support the robustness and generalizability of SGE as a functional assay for cancer susceptibility genes.

Importantly, integration of SGE results with ACMG criteria further improved clinical interpretability. Among the 88 VUS, 6 were reclassified as pathogenic and 64 as benign, substantially reducing diagnostic uncertainty. However, discrepant classifications were observed in a subset of variants, underscoring the necessity of incorporating multiple lines of evidence for accurate interpretation. In addition, pedigree analyses provided independent support for pathogenicity, with several functionally pathogenic variants associated with early-onset disease, bilateral BC, and familial clustering of BC or OC. These findings highlight the value of combining functional genomics with clinical and familial data to refine risk stratification. Recently, Shin et al. evaluated concordance of two SGE assays and available functional evidence for clinical variant classification [34]. Our work additionally analyzed correlations between SGE-based classifications, clinical phenotypes and familial cancer clustering in a large unselected breast cancer cohort. Together, these two studies validate the clinical value of consistent SGE evidence.

The DNA-binding domain (DBD) of BRCA2 is central to its tumor-suppressor function and represents a clinically important hotspot for missense variants [35,36]. Disruption of this region may compromise DNA repair [37]. Functionally pathogenic variants such as *c.7857G>C*(p.W2619C) and *c.9155G>A*(p.R3052Q) are located within the *BRCA2* C-terminal DNA-binding domain (DBD) and may affect conserved residues. The non-conservative amino acid substitutions are predicted to impair protein stability or DNA-binding affinity based on structural and evolutionary conservation analyses [38,39]. Indeed, definitive pathogenic classification for variants still requires further validation through clinical evidence and complementary functional experiments.

Beyond *BRCA2*, SGE offers a scalable platform for comprehensive functional interrogation of nearly all possible single-nucleotide variants within a gene [40,41,42]. Its ability to generate high-resolution, quantitative functional maps provides a distinct advantage over traditional approaches and has demonstrated high sensitivity and specificity in multiple validation studies [31,32,43,44]. As such, SGE has the potential to substantially reduce the burden of VUS in clinical genetic testing and improve the precision of genetic counseling and risk assessment [20,45].

Several limitations should be acknowledged. First, the number of carriers with functionally pathogenic variants was relatively small, which may limit statistical power. Second, comprehensive co-segregation analyses were not available for all families. Third, variants outside the SGE-targeted regions could not be evaluated. Future studies should aim to expand SGE coverage to the full coding region of *BRCA2* and more genes, include larger and more diverse cohorts, and incorporate longitudinal follow-up to assess clinical outcomes. In addition, the relationship between SGE-derived functional status and therapeutic response—particularly to PARP inhibitors and platinum-based chemotherapy—warrants further investigation.

## 5. Conclusions

In summary, SGE provides robust and reliable functional evidence for *BRCA2* VUS classification. Functionally pathogenic *BRCA2* variants are associated with distinct adverse clinical phenotypes similar to canonical pathogenic mutations. Integration of SGE with clinicopathological and pedigree data improves the accuracy and clinical actionability of variant interpretation. Our findings support the broader implementation of SGE in routine genetic testing to advance precision prevention and management of hereditary breast cancer.

## Figures and Tables

**Table 1:** Functional classification and carrier counts of 88 *BRCA2* VUS.

Functionally Pathogenic		VUS		Functionally Benign	
Variants	Number of Carriers	Variants	Number of Carriers	Variants	Number of Carriers
*c.7670C>T*(p.A2557V)	1	*c.7563C>G*(p.I2521M)	1	*c.7481G>A*(p.R2494Q)	1
*c.7766C>T*(p.P2589L)	1	*c.8596T>G*(p.F2866V)	1	*c.7488G>C*(p.K2496N)	3
*c.7786G>A*(p.G2596R)	1	*c.8861C>G* *(p.S2954C)	1	*c.7507G>A*(p.V2503I)	1
*c.7796A>G*(p.E2599G)	1	*c.8944A>C*(p.K2982Q)	2	*c.7522G>A*(p.G2508S)	44
*c.7857G>C*(p.W2619C)	1	*c.8961G>T* *(p.=)	1	*c.7540A>G*(p.K2514E)	1
*c.7871A>G*(p.Y2624C)	1	*c.8971C>T*(p.R2991C)	6	*c.7561A>G*(p.I2521V)	1
*c.7888A>G*(p.K2630E)	1	*c.9104A>C*(p.Y3035S)	1	*c.7601C>T*(p.A2534V)	3
*c.7967T>C*(p.L2656P)	1			*c.7603T>C*(p.C2535R)	3
*c.7978T>C* *(p.Y2660H)	1			*c.7628A>G*(p.Y2543C)	1
*c.8165C>T*(p.T2722I)	3			*c.7646G>A* *(p.C2549Y)	1
*c.8183T>G* *(p.V2728G)	1			*c.7648A>G*(p.I2550V)	1
*c.8258T>G* *(p.L2753R)	1			*c.7740G>T*(p.Q2580H)	1
*c.9155G>A*(p.R3052Q)	2			*c.7771A>G*(p.N2591D)	1
*c.9302T>C*(p.L3101P)	1			*c.7797A>G*(p.=)	1
*c.9625C>A*(p.P3209T)	3			*c.7805+4C>T*(p.?)	1
				*c.7825G>A*(p.G2609S)	1
				*c.7828G>A*(p.V2610M)	3
				*c.7862A>G*(p.Y2621C)	1
				*c.7901T>A*(p.M2634K)	5
				*c.7910C>T*(p.A2637V)	3
				*c.7965A>C* *(p.Q2655H)	1
				*c.7976+3A>G*(p.?)	1
				*c.7985C>T*(p.T2662M)	1
				*c.8009C>G*(p.S2670W)	3
				*c.8090G>A*(p.S2697N)	8
				*c.8092G>A*(p.A2698T)	9
				*c.8109T>C*(p.=)	1
				*c.8215G>A*(p.V2739I)	1
				*c.8299C>T*(p.P2767S)	1
				*c.8356G>A*(p.A2786T)	2
				*c.8359C>T*(p.R2787C)	4
				*c.8413T>C*(p.=)	1
				*c.8417C>T*(p.S2806L)	1
				*c.8458G>A* *(p.V2820I)	1
				*c.8471G>A*(p.R2824K)	1
				*c.8474C>A*(p.A2825E)	4
				*c.8518A>G*(p.I2840V)	2
				*c.8541A>G*(p.=)	1
				*c.8681A>C* *(p.Q2894P)	2
				*c.8682A>C* *(p.Q2894H)	2
				*c.8750T>C*(p.L2917P)	1
				*c.8796C>G* *(p.H2932Q)	2
				*c.8804T>C* *(p.M2935T)	1
				*c.8917C>G*(p.R2973G)	2
				*c.8918G>A*(p.R2973H)	1
				*c.9033T>G* *(p.=)	1
				*c.9096A>G*(p.=)	1
				*c.9106C>G* *(p.* *Q* *3036* *E* *)*	2
				*c.9116C>T*(p.P3039L)	3
				*c.9228A>G*(p.=)	1
				*c.9257*–*8C>T*(p.?)	1
				*c.9257*–*9C>T* *(p.?)	1
				*c.9275A>G*(p.Y3092C)	3
				*c.9275A>T*(p.Y3092F)	1
				*c.9286G>A*(p.E3096K)	1
				*c.9296A>G*(p.N3099S)	1
				*c.9309A>G*(p.I3103M)	1
				*c.9335A>G*(p.D3112G)	1
				*c.9337A>T*(p.I3113F)	1
				*c.9367A>G*(p.S3123G)	1
				*c.9383G>A*(p.R3128Q)	1
				*c.9422G>C* *(p.G3141A)	1
				*c.9501+6G>A*(p.?)	2
				*c.9583A>G*(p.T3195A)	1
				*c.9587A>G* *(p.K3196R)	1
				*c.9605C>G* *(p.P3202R)	1

VUS, variant of uncertain significance; RefSeq for *BRCA2*: NM_000059.4; “*” indicates novel variants that had not been reported in disease databases (the deadline for ClinVar is 30 May 2025). The 88 variants were classified according to the results of saturation genome editing; “Number of carriers” indicates the number of individuals carrying each variant.

**Table 2:** Clinicopathological characteristics among *BRCA2* germline variant carriers.

Characteristics	Pathogenic Variant Carriers		Functionally Pathogenic VUS Carriers		Functionally Benign VUS Carriers		Non-Carriers		P1	P2	P3	P4
	(*n* = 506)		(*n* = 20)		(*n* = 154)		(*n* = 13,535)					
	No.	%	No.	%	No.	%	No.	%				
Age at BC diagnosis, years												
Mean ± SD	47.3 ± 10.5		48.2 ± 9.4		49.9 ± 10.5		51.3 ± 11.3		<0.001	0.15	0.12	0.69
≤40 years	144	28.5	3	15.0	24	15.6	2256	16.7	<0.001	1.00	0.80	0.29
>40 years	362	71.5	17	85.0	130	84.4	11,279	83.3				
Family history of malignant tumor									<0.001	0.002	0.44	0.32
Yes	258	51.0	13	65.0	43	27.9	4214	31.1				
No	248	49.0	7	35.0	111	72.1	9321	68.9				
Family history of breast or ovarian cancer									<0.001	0.002	0.97	1.00
Yes	177	35.0	7	35.0	16	10.4	1352	10.0				
No	329	65.0	13	65.0	138	89.6	12,183	90.0				
Bilateral breast cancer									<0.001	0.085	0.28	1.00
Yes	61	12.1	2	10.0	6	3.9	330	2.4				
No	445	87.9	18	90.0	148	96.1	13,205	97.6				
Tumor size									0.10	0.62	0.70	0.81
≤2 cm	180	36.3	7	36.8	58	38.9	5284	41.1				
>2–5 cm	281	56.7	10	52.7	83	55.7	6750	52.5				
>5 cm	35	7.0	2	10.5	8	5.4	828	6.4				
Unknown	10		1		5		673					
ER status									<0.001	0.29	0.60	0.039
Negative	96	19.0	8	40.0	44	29.1	3547	26.9				
Positive	408	81.0	12	60.0	107	70.9	9645	73.1				
Unknown	2		0		3		343					
PR status									<0.001	0.051	1.00	0.005
Negative	124	24.6	11	55.0	49	32.5	4182	32.1				
Positive	380	75.4	9	45.0	102	67.5	8855	67.9				
Unknown	2		0		3		498					
HER2 status									<0.001	0.59	0.91	0.48
Negative	428	87.7	15	83.3	102	73.4	9073	74.2				
Positive	60	12.3	3	16.7	37	26.6	3162	25.8				
Unknown	18		2		15		1300					
Lymph nodes status									0.21	0.34	0.86	0.50
Negative	274	55.0	8	44.4	88	59.5	7411	58.4				
Positive	220	45.0	10	55.6	60	40.5	5280	41.6				
Unknown	12		2		6		844					

**Non-carriers:** Patients carrying *BRCA2* benign variants or without any *BRCA2* variant. ***p* values:** P1, *BRCA2* pathogenic variant carriers versus non-carriers; P2, functionally pathogenic VUS carriers versus non-carriers; P3, functionally benign VUS carriers versus non-carriers; P4, pathogenic variant carriers versus functionally pathogenic VUS carriers. **Abbreviations:** BC, breast cancer; SD, standard deviation; ER, estrogen receptor; PR, progesterone receptor; HER2, human epidermal growth factor receptor 2; VUS, variant of uncertain significance. **Statistical note:** Pairwise comparisons were adjusted using the Bonferroni correction (*p* < 0.0125).

## Data Availability

The data supporting the findings of this study are available from the corresponding author upon reasonable request.

## Supplementary Materials

The following supporting information can be downloaded at: https://www.mdpi.com/article/10.3390/curroncol33090521/s1, Table S1. Comprehensive annotation and SGE-based reclassification of 88 *BRCA2* variants of uncertain significance. Table S2. Classification Status of 88 *BRCA2* Variants of Uncertain Significance (VUS) Loci in Two SGE Experiments. Table S3. Clinicopathological characteristics among *BRCA2* germline variant carriers (Based on SGE and ACMG).
