# Supplementary material for: Reclassification of BRCA2 Variants of Uncertain Significance Using Saturation Genome Editing Combined with Clinical Phenotypes in Breast Cancer
Source: Curr Oncol. 2026 Aug 31;33(9):521. doi: 10.3390/curroncol33090521 (PMC13605375; doi:10.3390/curroncol33090521)
Supplement: Supplementary file 1 [file curroncol-33-00521-s001.zip › Supplementary Table S1.docx]

**Supplementary Table S1. Comprehensive annotation and SGE-based reclassification of 88 *BRCA2* variants of uncertain significance.**

| **HGVS  Nucleotide (Protein)** | **Exon** | **Mutation Type** | **Domain** | **Novel variants** | **Population Databases** | | | | | **Computational predictions** | | | | **Other functional experiments** | **ClinVar (2025.5)** | **Reclassification** |
| --- | --- | --- | --- | --- | --- | --- | --- | --- | --- | --- | --- | --- | --- | --- | --- | --- |
|  |  |  |  |  | **1000 Genomes** | **1000 Genomes _EAS** | **esp6500_all** | **ExAC** | **ExAC _EAS** | **SIFT score (pred)** | **Polyphen2_HDIV score (pred)** | **Polyphen2_HVAR score (pred)** | **Mutation Taster score (pred)** |  |  |  |
| c.7481G>A  (p.R2494Q) | Exon15 | Missense | DBD | No |  |  |  |  |  | 0.03(D) | 0.998(D) | 0.403(B) | 1(D) | Guidugli L.et al(2018);  Richardson ME et al(2021) | Conflicting classifications of pathogenicity | Functionally benign |
| c.7488G>C (p.K2496N) | Exon15 | Missense | DBD | No | . | . | . | 0.000025 | 0.000347 |  |  |  |  | . | Uncertain significance | Functionally benign |
| c.7507G>A (p.V2503I) | Exon15 | Missense | DBD | No | . | . | . | 0.000008263 | 0 | 1(T) | 0.001(B) | 0.002(B) | 1(N) | . | Conflicting classifications of pathogenicity | Functionally benign |
| c.7522G>A (p.G2508S) | exon15 | Missense | DBD | No | . | . | . | 0.000107. | 0.001505 | . | . | . | . | Shimelis H et al.(2017);  Mesman RLS et al.(2018);  Guidugli L et al.(2018);  Ikegami M et al.(2020） | Conflicting classifications of pathogenicity | Functionally benign |
| c.7540A>G  (p.K2514E) | exon15 | Missense | DBD | No |  |  |  |  |  | 0.01(D) | 1(D) | 0.985(D) | 1(D) |  | Conflicting classifications of pathogenicity | Functionally benign |
| c.7561A>G (p.I2521V) | exon15 | Missense | DBD | No | . | . | . | . | . | 0.007(D) | 0.053(B) | 0.013(B) | 0.709(N) | . | Conflicting classifications of pathogenicity. | Functionally benign |
| c.7563C>G (p.I2521M) | exon15 | Missense | DBD | No | . | . | . | . | . | 0.004(D) | 0.978(D) | 0.775(P) | 0.526(D) | . | .Uncertain significance | VUS |
| c.7601C>T (p.A2534V) | exon15 | Missense | DBD | No | 0.000599 | 0 | 0.000077 | 0.0001 | 0 |  |  |  |  | . | Conflicting classifications of pathogenicity | Functionally benign |
| c.7603T>C (p.C2535R) | exon15 | Missense | DBD | No | . | . | . | . | . |  |  |  |  | . | Uncertain significance | Functionally benign |
| c.7628A>G (p.Y2543C) | exon15 | Missense | DBD | No | . | . | . | 0.00004145 | 0 | 0.12(T) | 0.278(B) | 0.12(B) | 0.926(D) | Caleca L et al.(2019);  Pouliot GP et al.(2019) | Conflicting classifications of pathogenicity | Functionally benign |
| c.7646G>A* (p.C2549Y) | exon15 | Missense | DBD | Yes | . | . | . | . |  | 0.025(D) | 0.714(P) | 0.159(B) | 1(N) | . |  | Functionally benign |
| c.7648A>G (p.I2550V) | exon15 | Missense | DBD | No | . | . | . | 0.00000826 | 0 | 0.388(T) | 0.001(B) | 0.001(B) | 1(N) | Park et al.,2017; Lee et al., 2010; Hayes et al., 2000 | Conflicting classifications of pathogenicity | Functionally benign |
| c.7670C>T (p.A2557V) | exon15 | Missense | DBD | No | . | . | 0.00000825 | 0.00001 | 0.001 |  |  |  |  | Lu et al.,2015; Zhang et al., 2017 | Uncertain significance | functionally pathogenic |
| c.7740G>T (p.Q2580H) | exon15 | Missense | DBD | No | . | . | . | . | . | 0.028(D) | 0.955(P) | 0.472(P) | 0.984(D) | Lee et al., 2010 | Conflicting classifications of pathogenicity | Functionally benign |
| c.7766C>T (p.P2589L) | exon15 | Missense | DBD | No | . | . | . | . | . |  |  |  |  | Lee et al., 2010;  Quaresima et al., 2006 | Uncertain significance | functionally pathogenic |
| c.7771A>G (p.N2591D) | exon15 | Missense | DBD | No | . | . | . | . | . |  |  |  |  | . | Conflicting classifications of pathogenicity. | Functionally benign |
| c.7786G>A (p.G2596R) | exon15 | Missense | DBD | No | . | . | . | 0.00000825 | 0 | 0(D) | 1(D) | 1(D) | 1(D) | Biswas K et al.(2020);  Guidugli L et al.(2018);  Hart SN et al.(2019) | Conflicting classifications of pathogenicity | functionally pathogenic |
| c.7796A>G (p.E2599G) | exon15 | Missense | DBD | No | . | . | . | . | . | 0.01(D) | 1(D) | 1(D) | 1(D) | Castells-Roca L et al(2021) | Conflicting classifications of pathogenicity | functionally pathogenic |
| c.7797A>G (p.=) | exon16 | Synonymous | DBD | Yes | . | . | . | . | . |  |  |  |  | . | Conflicting classifications of pathogenicity | Functionally benign |
| c.7805+4C>T (p.?) | exon16 | Splice | DBD | No | . | . | . | 0.000008 | 0 |  | . | . | . | . | Uncertain significance | Functionally benign |
| c.7825G>A (p.G2609S) | exon17 | Missense | DBD | No | . | . | . |  |  | 0(D) | 1(D) | 1(D) | 1(D) | . | Uncertain significance | Functionally benign |
| c.7828G>A (p.V2610M) | exon17 | Missense | DBD | No | . | . | . | . | . |  |  |  |  | Whiley PJ et al.(2014) | Uncertain significance | Functionally benign |
| c.7857G>C (p.W2619C) | exon17 | Missense | DBD | No |  | . | . |  |  | 0(D) |  |  | 1(D) | . | Conflicting classifications of pathogenicity. | functionally pathogenic |
| c.7862A>G (p.Y2621C) | exon17 | Missense | DBD | No | . | . | . | . | . | 0.09(T) | 0.638(P) | 0.316(B) | 0.93(N) | . | Conflicting classifications of pathogenicity | Functionally benign |
| c.7871A>G (p.Y2624C) | exon17 | Missense | DBD | No | . | . | . | 0.000008 | 0 |  |  |  |  | Hart SN et al.(2019);  Richardson ME et al.(2021) | Conflicting classifications of pathogenicity | functionally pathogenic |
| c.7888A>G (p.K2630E) | exon17 | Missense | DBD | No | . | . | . | . | . | 0.001(D) | 1(D) | 1(D) | 1(D) | . | Uncertain significance | functionally pathogenic |
| c.7901T>A (p.M2634K) | exon17 | Missense | DBD | No | . | . | . | . | . |  |  |  |  |  | Conflicting classifications of pathogenicity | Functionally benign |
| c.7910C>T (p.A2637V) | exon17 | Missense | DBD | No | . | . | . |  |  |  |  |  |  | . | Conflicting classifications of pathogenicity | Functionally benign |
| c.7965A>C*  (p.Q2655H) | exon17 | Missense | DBD | Yes |  |  |  |  |  | 0(D) | 1(D) | 1(D) | 0.999(D) |  |  | Functionally benign |
| c.7967T>C (p.L2656P) | exon17 | Missense | DBD | No | . | . | . | . | . |  |  |  |  | . | Uncertain significance | functionally pathogenic |
| c.7976+3A>G (p.?) | exon17 | Splice | DBD | Yes | . | . | . | . | . |  |  |  |  | . | Uncertain significance. | Benign strong |
| c.7978T>C* (p.Y2660H) | exon18 | Missense | DBD | Yes | . | . | . | . | . | 0(D) | 1(D) | 1(D) | 1(D) | . | . | functionally pathogenic |
| c.7985C>T (p.T2662M) | exon18 | Missense | DBD | No | . | . | . |  |  |  |  |  |  | Richardson ME et al.(2021);  Hart SN et al.(2019) | Conflicting classifications of pathogenicity | Functionally benign |
| c.8009C>G (p.S2670W) | exon18 | Missense | DBD | No | . | . | . | . | . | 0(D) |  |  | 1(D) | Fraile-Bethencourt Eet al.(2017) | Conflicting classifications of pathogenicity | Functionally benign |
| c.8090G>A (p.S2697N) | exon18 | Missense | DBD | No | . | . | 0.000077 | 0.000049 | 0.000347 |  |  |  |  | . | Conflicting classifications of pathogenicity | Functionally benign |
| c.8092G>A  （p.A2698T） | exon18 | Missense | DBD | No | 0 | 0 |  | 0.00003 | 0.000231 |  |  |  |  |  | Conflicting classifications of pathogenicity | Functionally benign |
| c.8109T>C  （p.=） | exon18 | Synonymous | DBD | Yes |  |  |  |  |  |  |  |  |  |  | Conflicting classifications of pathogenicity | Functionally benign |
| c.8165C>T  （p.T2722I） | exon18 | Missense | DBD | No |  |  |  |  |  |  |  |  |  | Richardson ME et alet al.(2021) | Conflicting classifications of pathogenicity | functionally pathogenic |
| c.8183T>G*（p.V2728G） | exon18 | Missense | DBD | Yes |  |  |  |  |  | 0(D) | 0.99(D) | 0.888(P) | 1(D) |  |  | functionally pathogenic |
| c.8215G>A（p.V2739I） | exon18 | Missense | DBD | No |  |  |  | 0.000016 | 0.000231 |  |  |  |  | Karchin R(2008);  Biswas K et al.(2020) | Conflicting classifications of pathogenicity | Functionally benign |
| c.8258T>G*  (p.L2753R) | exon18 | Missense | DBD | Yes |  |  |  |  |  |  |  |  |  |  |  | functionally pathogenic |
| c.8299C>T（p.P2767S） | exon18 | Missense | DBD | No |  |  |  |  |  |  |  |  |  | Hu C et al.(2022) | Conflicting classifications of pathogenicity | Functionally benign |
| c.8356G>A（p.A2786T） | exon19 | Missense | DBD | No |  |  |  | 0.000058 | 0.000809 |  |  |  |  | Consortium et al.(2021);  Sullivan T et al.(2021);  Guidugli L et al.(2018) | Conflicting classifications of pathogenicity | Functionally benign |
| c.8359C>T  (p.R2787C) | exon19 | Missense | DBD | No |  |  |  |  |  |  |  |  |  | Guidugli L et al.(2018);  Hart SN et al.(2019);  Parsons MT et al.(2019) | Conflicting classifications of pathogenicity | Functionally benign |
| c.8413T>C  （p.=） | exon19 | Synonymous | DBD | Yes |  |  |  |  |  |  |  |  |  |  | Conflicting classifications of pathogenicity | Functionally benign |
| c.8417C>T（p.S2806L） | exon19 | Missense | DBD | No |  |  |  |  |  |  |  |  |  |  | Conflicting classifications of pathogenicity | Functionally benign |
| c.8458G>A*（p.V2820I） | exon19 | Missense | DBD | Yes |  |  |  | 0.0002 | 0 | 0(D) | 1(D) | 0.967(D) | 1(D) | Richardson ME et al.(2021);  Hu C et al.(2022) | Conflicting classifications of pathogenicity | Functionally benign |
| c.8471G>A  (p.R2824K) | exon19 | Missense | DBD | Yes |  |  |  |  |  |  |  |  |  |  |  | Functionally benign |
| c.8474C>A  (p.A2825E) | exon19 | Missense | DBD | No |  |  |  |  |  |  |  |  |  |  | Conflicting classifications of pathogenicity | Functionally benign |
| c.8518A>G（p.I2840V） | exon20 | Missense | DBD | No |  |  |  | 0.000016 | 0 |  |  |  |  | Biswas K et al.(2023) | Conflicting classifications of pathogenicity | Functionally benign |
| c.8541A>G  （p.=） | exon20 | synonymous | DBD | Yes |  |  |  |  |  |  |  |  |  |  | Conflicting classifications of pathogenicity | Functionally benign |
| c.8596T>G（p.F2866V） | exon20 | Missense | DBD | No |  |  |  |  |  | 0.07(T) | 0.925(P) | 0.572(P) | 0.995(N) |  | Uncertain significance | VUS |
| c.8681A>C*（p.Q2894P） | exon21 | Missense | DBD | Yes |  |  |  |  |  |  |  |  |  |  |  | Functionally benign |
| c.8682A>C*  (p.Q2894H) | exon21 | Missense | DBD | Yes |  |  |  |  |  |  |  |  |  |  |  | Functionally benign |
| c.8750T>C（p.L2917P） | exon21 | Missense | DBD | No |  |  |  |  |  | 0.001(D) | 0.998(D) | 0.816(P) | 1(D) |  | Conflicting classifications of pathogenicity | Functionally benign |
| c.8796C>G*  (p.H2932Q) | exon22 | Missense | DBD | Yes |  |  |  |  |  |  |  |  |  |  |  | Functionally benign |
| c.8804T>C*（p.M2935T） | exon22 | Missense | DBD | Yes |  |  |  |  |  |  |  |  |  |  |  | Functionally benign |
| c.8861C>G*（p.S2954C） | exon22 | Missense | DBD | Yes |  |  |  |  |  | 0.129(T) | 0.91(P) | 0.197(（B) | 1(N) |  |  | VUS |
| c.8917C>G（p.R2973G） | exon22 | Missense | DBD | No |  |  |  | 0.00000831 | 0.0001 | 0.022(D) | 1(D) | 0.999(D) | 0.826(N) |  | Uncertain significance | Functionally benign |
| c.8918G>A（p.R2973H） | exon22 | Missense | DBD | No |  |  | 0.0002 | 0.0000416 | 0.0002 | 0.005(D) | 1(D) | 0.998(D) | 0.719(N) | Hart SN et al.(2019);  Richardson ME et al.(2021) | Conflicting classifications of pathogenicity | Functionally benign |
| c.8944A>C（p.K2982Q） | exon22 | Missense | DBD | No |  |  |  | 0.000058 | 0 |  |  |  |  |  | Conflicting classifications of pathogenicity | VUS |
| c.8961G>T*  （p.=） | exon22 | synonymous | DBD | Yes |  |  |  |  |  |  |  |  |  |  |  | VUS |
| c.8971C>T（p.R2991C） | exon23 | Missense | DBD | No |  |  |  | 0.000008 | 0 |  |  |  |  | Hart SN et al.(2019) | Conflicting classifications of pathogenicity | VUS |
| c.9033T>G*  (p.=) | exon23 | Missense | DBD | Yes |  |  |  |  |  |  |  |  |  |  |  | Functionally benign |
| c.9096A>G  （p.=） | exon23 | Synonymous | DBD | Yes |  |  |  |  |  |  |  |  |  |  | Conflicting classifications of pathogenicity | Functionally benign |
| c.9104A>C（p.Y3035S） | exon23 | Missense | DBD | No |  |  | 0.000077 | 0.0000515 | 0 | 0(D) | 1(D) | 0.986(D) | 0.989(D) | Théry JC et al.(2011);  Wai HA et al.(2020);  Guidugli L et al.(2018);  Ikegami M et al.(2020) | Conflicting classifications of pathogenicity | VUS |
| c.9106C>G（p.Q3036E） | exon23 | Missense | DBD | No | 0.0002 | 0.001 |  | 0.000034 | 0.000492 |  |  |  |  | sullivan et al(2021) | Conflicting classifications of pathogenicity | Functionally benign |
| c.9116C>T  (p.P3039L) | exon23 | Missense | DBD | No |  |  |  |  | 0 |  |  |  |  | Caux-Moncoutier V et al.(2009);  Houdayer C et al.(2012);  Menéndez M et al.(2012);  Colombo M et al.(2013);  Guidugli L et al.(2018);  Hart SN et al.(2019);  Richardson ME et al.(2021);  Rodríguez-Balada M et al.(2016) | Conflicting classifications of pathogenicity | Functionally benign |
| c.9155G>A（p.R3052Q） | exon24 | Missense | DBD | No |  |  |  | 0.0000331 | 0 | 0(D) | 1(D) | 1(D) | 1(D) |  | Conflicting classifications of pathogenicity | functionally pathogenic |
| c.9228A>G  （p.=） | exon24 | Synonymous | DBD | Yes |  |  |  |  |  |  |  |  |  |  | Conflicting classifications of pathogenicity | Functionally benign |
| c.9257-8C>T  (p.?) | exon24 | Splice | DBD | No |  |  |  | 0.00001 |  |  |  |  |  |  | Conflicting classifications of pathogenicity | Functionally benign |
| c.9257-9C>T*  (p.?) | exon24 | Splice | DBD | Yes |  |  |  |  |  |  |  |  |  |  |  | Functionally benign |
| c.9275A>G（p.Y3092C） | exon25 | Missense | DBD | No |  |  |  | 0.0000609 | 0 | 0(D) | 1(D) | 1(D) | 1(D) |  | Conflicting classifications of pathogenicity | Functionally benign |
| c.9275A>T（p.Y3092F） | exon25 | Missense | DBD | No |  |  |  | 0.00000869 | 0 | 0(D) | 1(D) | 1(D) | 1(D) | Caux-Moncoutier V et al.(2009); Houdayer C et al.(2012);  Guidugli L et al.(2013) | Conflicting classifications of pathogenicity | Functionally benign |
| c.9286G>A  (p.E3096K) | exon25 | Missense | DBD | No |  |  |  | 0.00000858 | 0 | 0(D) | 1(D) | 0.97(D) | 1(D) | Ikegami M et al.(2020) | Conflicting classifications of pathogenicity | Functionally benign |
| c.9296A>G  p.(N3099S) | exon25 | Missense | DBD | No |  |  |  |  |  |  |  |  |  |  | Conflicting classifications of pathogenicity | Functionally benign |
| c.9302T>C（p.L3101P） | exon25 | Missense | DBD | No |  |  |  |  |  |  |  |  |  | Hu C et al.(2022);  Ikegami M et al.(2020);  Hu C et al.(2024) | Conflicting classifications of pathogenicity | functionally pathogenic |
| c.9309A>G（p.I3103M） | exon25 | Missense | DBD | No |  |  |  |  |  | 0.002(D) | 0.991(D) | 0.947(D) | 0.986(N) |  | Conflicting classifications of pathogenicity | Functionally benign |
| c.9335A>G（p.D3112G） | exon25 | Missense | DBD | No |  |  |  |  |  |  |  |  |  |  | Uncertain significance | Functionally benign |
| c.9337A>T（p.I3113F） | exon25 | Missense | DBD | No |  |  |  |  |  | 0.034(D) | 0.125(B) | 0.104(B) | 1(N) |  | Uncertain significance | Functionally benign |
| c.9367A>G（p.S3123G） | exon25 | Missense | DBD | No |  |  |  | 0.000008249 | 0 | 0（D) |  |  | 0.977(D) | Hu C et al.(2022);  Hart SN et al.(2019) | Conflicting classifications of pathogenicity | Functionally benign |
| c.9383G>A（p.R3128Q)） | exon25 | Missense | DBD | No |  |  |  | 0.000008 | 0.000116 |  |  |  |  | Guo Q et al.(2023) | Conflicting classifications of pathogenicity | Functionally benign |
| c.9422G>C*（p.G3141A） | exon25 | Missense | DBD | Yes |  |  |  |  |  | 0.02(D) | 0.999(D) | 0.977(D) | 1(D) |  |  | Functionally benign |
| c.9501+6G>A  (p.?) | exon25 | Splice | DBD | Yes |  |  |  |  |  |  |  |  |  |  | Conflicting classifications of pathogenicity | Functionally benign |
| c.9583A>G（p.T3195A） | exon25 | Missense | DBD | No |  |  |  | 0.000008 | 0 |  |  |  |  |  | Conflicting classifications of pathogenicity | Functionally benign |
| c.9587A>G*  (p.K3196R) | exon25 | Missense | DBD | Yes |  |  |  |  |  |  |  |  |  |  |  | Functionally benign |
| c.9605C>G*（p.P3202R） | exon26 | Missense | DBD | Yes |  |  |  |  |  |  |  |  |  |  |  | Functionally benign |
| c.9625C>A（p.P3209T） | exon26 | Missense | DBD | No |  |  |  |  |  | 0.011(D) | 0.079(B) | 0.018(B) | 1(N) |  | Uncertain significance | functionally pathogenic |

RefSeq for *BRCA2*: NM_000059.4. “*” indicates novel variants that had not been reported in disease databases (The deadline for ClinVar is May 30, 2025.); DBD, DNA-binding domain; SGE, saturation genome editing.
