# Supplementary material for: Reclassification of BRCA2 Variants of Uncertain Significance Using Saturation Genome Editing Combined with Clinical Phenotypes in Breast Cancer
Source: Curr Oncol. 2026 Aug 31;33(9):521. doi: 10.3390/curroncol33090521 (PMC13605375; doi:10.3390/curroncol33090521)
Supplement: Supplementary file 1 [file curroncol-33-00521-s001.zip › Supplementary Table S3.docx]

**Supplementary Table S3. Clinicopathological characteristics among *BRCA2* germline variant carriers (Based on SGE and ACMG).**

| Characteristics | Pathogenic variant  carriers | | Functionally pathogenic  VUS carriers | | Functionally benign  VUS carriers | | Non-carriers | | P1 | P2 | P3 | P4 |
| --- | --- | --- | --- | --- | --- | --- | --- | --- | --- | --- | --- | --- |
|  | (n=506) | | (n=9) | | (n=151) | | (n=13535) |  |  |  |  |  |
|  | No. | % | No. | % | No. | % | No. | % |  |  |  |  |
| Age at BC diagnosis, years | | | | | | | | | | | | |
| Mean ± SD | 47.3$\text{±}$10.5 |  | 46.8$\text{±}$6.0 |  | 50.1±10.5 |  | 51.3$\text{±}$11.3 |  | <0.001 | 0.057 | 0.18 | 0.82 |
| ≤40 years | 144 | 28.5 | 1 | 11.1 | 23 | 15.2 | 2256 | 16.7 | <0.001 | 1.00 | 0.72 | 0.46 |
| >40 years | 362 | 71.5 | 8 | 88.9 | 128 | 84.8 | 11279 | 83.3 |  |  |  |  |
| Family history of malignant tumor | | | | | | | | | <0.001 | 0.030 | 0.54 | 0.51 |
| Yes | 258 | 51.0 | 6 | 66.7 | 43 | 28.5 | 4214 | 31.1 |  |  |  |  |
| No | 248 | 49.0 | 3 | 33.3 | 108 | 71.5 | 9321 | 68.9 |  |  |  |  |
| Family history of breast or ovarian cancer | | | | | | | | | <0.001 | 0.008 | 0.91 | 0.73 |
| Yes | 177 | 35.0 | 4 | 44.4 | 16 | 10.6 | 1352 | 10.0 |  |  |  |  |
| No | 329 | 65.0 | 5 | 55.6 | 135 | 89.4 | 12183 | 90.0 |  |  |  |  |
| Bilateral breast cancer | | | | | | | | | <0.001 | 0.20 | 0.28 | 1.00 |
| Yes | 61 | 12.1 | 1 | 11.1 | 6 | 4.0 | 330 | 2.4 |  |  |  |  |
| No | 445 | 87.9 | 8 | 88.9 | 145 | 96.0 | 13205 | 97.6 |  |  |  |  |
| Tumor size | | | | | | | | | 0.10 | 0.86 | 0.74 | 1.00 |
| ≤2cm | 180 | 36.3 | 3 | 33.3 | 57 | 39.0 | 5284 | 41.1 |  |  |  |  |
| >2-5cm | 281 | 56.7 | 6 | 66.7 | 81 | 55.5 | 6750 | 52.5 |  |  |  |  |
| >5cm | 35 | 7.0 | 0 | 0.0 | 8 | 5.5 | 828 | 6.4 |  |  |  |  |
| Unknown | 10 |  | 0 |  | 5 |  | 673 |  |  |  |  |  |
| ER status | | | | | | | | | <0.001 | 0.065 | 0.50 | 0.017 |
| Negative | 96 | 19.0 | 5 | 55.6 | 44 | 29.7 | 3547 | 26.9 |  |  |  |  |
| Positive | 408 | 81.0 | 4 | 44.4 | 104 | 70.3 | 9645 | 73.1 |  |  |  |  |
| Unknown | 2 |  | 0 |  | 3 |  | 343 |  |  |  |  |  |
| PR status | | | | | | | | | <0.001 | 0.007 | 0.86 | 0.001 |
| Negative | 124 | 24.6 | 7 | 77.8 | 49 | 33.1 | 4182 | 32.1 |  |  |  |  |
| Positive | 380 | 75.4 | 2 | 22.2 | 99 | 66.9 | 8855 | 67.9 |  |  |  |  |
| Unknown | 2 |  | 0 |  | 3 |  | 498 |  |  |  |  |  |
| HER2 status | | | | | | | | | <0.001 | 1.00 | 0.80 | 0.26 |
| Negative | 428 | 87.7 | 6 | 75.0 | 99 | 72.8 | 9073 | 74.2 |  |  |  |  |
| Positive | 60 | 12.3 | 2 | 25.0 | 37 | 27.2 | 3162 | 25.8 |  |  |  |  |
| Unknown | 18 |  | 1 |  | 15 |  | 1300 |  |  |  |  |  |
| Lymph nodes status | | | | | | | | | 0.21 | 1.00 | 0.89 | 1.00 |
| Negative | 274 | 55.0 | 5 | 62.5 | 86 | 59.3 | 7411 | 58.4 |  |  |  |  |
| Positive | 220 | 45.0 | 3 | 37.5 | 59 | 40.7 | 5280 | 41.6 |  |  |  |  |
| Unknown | 12 |  | 1 |  | 6 |  | 844 |  |  |  |  |  |

**Non-carriers:** Non-carriers refer to patients carrying *BRCA2* benign variants or patients not carrying any *BRCA2* variant.

**P values:** P1, *BRCA2* pathogenic variant carriers versus non-carriers; P2, functionally pathogenic VUS carriers versus non-carriers; P3, functionally benign VUS carriers versus non-carriers; P4, pathogenic variant carriers versus functionally pathogenic VUS carriers.

**Abbreviations:** BC, breast cancer; SD, standard deviation; ER, estrogen receptor; PR, progesterone receptor; HER2, human epidermal growth factor receptor 2; VUS, variant of uncertain significance.
